# cnvCurator: an interactive visualization and editing tool for somatic copy number variations

**DOI:** 10.1186/s12859-015-0766-y

**Published:** 2015-10-15

**Authors:** Lingnan Ma, Maochun Qin, Biao Liu, Qiang Hu, Lei Wei, Jianmin Wang, Song Liu

**Affiliations:** Department of Biostatistics and Bioinformatics, Roswell Park Cancer Institute, Buffalo, NY 14263 USA; Department of Mathematics, University at Buffalo, Buffalo, NY 14260 USA; College of Engineering, University of Michigan, Ann Arbor, MI 48109 USA

**Keywords:** Somatic copy number variation, Next generation sequencing, Cancer genome analysis, Interactive data viewer

## Abstract

**Background:**

One of the most important somatic aberrations, copy number variations (CNVs) in tumor genomes is believed to have a high probability of harboring oncotargets. Detection of somatic CNVs is an essential part of cancer genome sequencing analysis, but the accuracy is usually limited due to various factors. A post-processing procedure including manual review and refinement of CNV segments is often needed in practice to achieve better accuracy.

**Results:**

cnvCurator is a user-friendly tool with functions specifically designed to facilitate the process of interactively visualizing and editing somatic CNV calling results. Different from other general genomics viewers, the index and display of CNV calling results in cnvCurator is segment central. It incorporates multiple CNV-specific information for concurrent, interactive display, as well as a number of relevant features allowing user to examine and curate the CNV calls.

**Conclusions:**

cnvCurator provides important and practical utilities to assist the manual review and edition of results from a chosen somatic CNV caller, such that curated CNV segments will be used for down-stream applications.

## Background

CNV was initially classified as gain or loss of a chromosome segment with a length greater than 1 kb, and then widened to include much smaller events (>50 bps) on accommodating the improved resolution of detection methods [1–3]. In cancer, CNV is one of the most important somatic aberrations, and has been identified as the driver event in many cancer types [1, 4, 5]. The widespread adoption of next-generation sequencing (NGS) provides an unprecedented opportunity to systematically screen for somatic CNVs in cancer genomes, and has been emerging as the primary means of interrogating the CNVs in recent investigations [6]. Accurate detection of CNVs from massive amounts of raw NGS data of the cancer genome requires sophisticated computational algorithms, with read depth, B Allele Frequency (BAF), split reads, and discordant read pairs derived from sequence read mapping as the primary input [7].

A number of computational methods have been developed to identify CNVs using NGS data [7, 8]. However, recent evaluations show each algorithm has its own strengths and weaknesses, and none of the CNV calling methods performed well in all situations [9]. The concordance of different CNV calling tools is especially low in real applications [10–12], suggesting that caution and care are needed to interpret and report the calling results, and additional post-processing might be needed to achieve maximum accuracy.

An ideal CNV report should accurately quantify the copy numbers in all genomic segments and delineate their breakpoints across the whole genome. In cancer study, CNV calling is particularly challenging due to tumor purity, heterogeneity, and aneuploidy [13–15]. Inaccurate segmentation could create false positive segments and miss true breakpoints, which will greatly affect downstream interpretation and application of CNV calls. Therefore, a post-processing procedure including manual review and curation of the CNV calling results is often required to reduce incorrect predictions in cancer genome analysis [16–18]. Manual review is a process to identify false segmentation, missing breakpoints, and incorrect copy number status by visually checking relevant information underlying the CNV calling, such as read depth information of both tumor and normal samples, BAF of germline heterozygous SNVs (single nucleotide variations) in tumor, and signatures of reads and read-pairs (e.g., soft-clipped reads and discordant read pairs) related to breakpoints at a segment boundary. After an error is identified, an edition process needs to be performed to update the CNV boundaries as well as the related quantitative information.

Although several CNV detection methods provide certain types of graphical output for inspection, none of them provides an interactive viewer for result review and curation. While existing viewers such as Integrative Genomics Viewer [19] have sophisticated interfaces for interactive visualization, they are not tailored to CNV-specific applications. Furthermore, they generally do not provide any functions to facilitate manual curation of CNV calling results. Here, we present cnvCurator, a platform-independent visualization and editing tool to facilitate manual inspection and curation of somatic CNV calling. Different from other general genomics viewers, cnvCurator incorporates multiple CNV-related information for concurrent, interactive display, as well as a number of practical features allowing user to examine and refine the CNV calls.

## Implementation

cnvCurator is a Java utility designated to provide an interactive platform for conveniently reviewing and curating CNV predictions. There are two major components: 1) segment-central index and display of CNV calls for the purpose of manual review, and 2) editing the problematic CNV segments identified from manual review.

### Segment-central index and display of CNV calls for manual review

The list of segments obtained from a given somatic CNV calling program will be indexed on the left of the main cnvCurator window. The segments can be generally classified as copy gain, loss or neutral (i.e., diploid normal without copy number variations), depending on the program’s threshold for signal difference between tumor and matched normal. We list all segments on the left of the main window so that users can not only review a segment of copy number gain or loss, but also examine whether a segment of copy neutral call might be false negative, or contain sub-segment of copy number gain/loss. For easy navigation, we have added a feature to display the segment of copy loss as blue, copy gain as red, and copy neutral as black.

For a given segment of interest, cnvCurator supports concurrent visualization of diverse CNV-related data types on the right of the main window, which include 1) the ideogram of currently displayed chromosome, 2) the genomic coordinates, 3) the sequencing read depth information for tumor and matched normal samples, 4) the logR ratio derived from read depth data, 5) BAF at germline SNV positions [20], 6) details of sequencing read alignment for tumor and matched normal samples, and 7) annotations of genomic features. These tracks provide both direct and indirect evidence from multiple angles to help the reviewer make decisions about the confidence of a given CNV call.

A number of navigating functions such as zoom in/out, moving around the genome, displaying detailed read alignment information, specifying alternative threshold for segment indexing, and changing of color scheme, are implemented in cnvCurator to assist CNV manual review and curation.

#### Navigate between segments

cnvCurator loads CNV segments into a panel docked at the left side of the application, and organizes the data into a tree structure with segments from the same chromosomes as leaves under the same node. By clicking any segment, the viewer switches to the corresponding genomic region with extra adjacent context displayed. The fraction of flanking regions can be specified by the user.

#### Display breakpoint windows

Real CNV boundaries are often associated with structural variations which can be detected by split reads or discordant read pairs [21–24]. Therefore, the presence of such reads can be used as supporting information to distinguish real CNVs from false calls caused by uneven sequencing read depth. In addition to the traditional alignment tracks which allow zoom-in/out visualization, cnvCurator provides advanced options to display such signatures in a user-friendly way. For example, once a CNV segment is selected, two pop-up windows for the two breakpoints (±100 bps by default) of the segment will be automatically displayed. Users can zoom-in/out the breakpoint pop-up windows, and simultaneously display multiple pop-up windows in adjacent regions to examine alternative breakpoints.

### Edit the problematic CNV segments identified from manual review

An important and unique feature of cnvCurator is the ability to dynamically refine the results during the manual review process and generate a set of manually curated CNV calls. Mis-segmentation is a common problem for CNV detection methods, which will greatly affect downstream interpretation and application of CNV calls. It could be the missing of true breakpoints, which could incorrectly merge two distinct segments into one or miss the genuine segments. It could also be introducing false breakpoints, which could incorrectly separate one segment into two parts, create segments with incorrect boundary, or create entirely false segments. Once the segmentation errors are spotted during the manual review, it would be handy to be able to fix them and have the segment list updated. cnvCurator provides several functionalities to assist the manual curation procedure, such as removing a false/spurious call, adding a missed/genuine segment, and correcting the breakpoint (s) of a segment with incorrect boundary. These can be achieved through merging two adjacent segments and/or splitting a segment. Merging adjacent segments is less complex, while splitting a segment requires knowing the exact location of the new breakpoint. Users can use cnvCurator to narrow down potential breakpoint location in the current window by identifying the position which minimizes the variations of logR ratio within the two segments. When there are multiple break points in the current window (segment), the user can recursively apply the splitting function within each sub-window (segment) to generate multiple new segments. We will implement the function of simultaneously splitting multiple breakpoints in the future version once we can find a solid statistical model for this problem. The user can adjust the range of the current window and examine supporting features (e.g., reads alignment signature) to refine the searching. The segment texts on the left of the main window will be automatically updated once the segment edition (i.e., through splitting and/or merging the segments) is performed, and we have provided an “undo” function to these operations in the software. The updated segment list can be saved to a file and reloaded in the segmentation panel. The detailed tutorial to perform CNV editing during manual review process can be found in the project website. It should be noted that the process of merging two adjacent segments and/or splitting a segment with cnvCurator is not automated, and these editing functions are provided to assist in the manual review process.

## Results

The inputs for cnvCurator include 1) the CNV calling results in segmented data file format [25] from a chosen somatic CNV caller 2) the read depth file in bigWig format for tumor and matched normal sample, separately, 3) the BAF file in bigWig format for tumor sample, and 4) the read alignment file in Binary Alignment/Map (BAM) format [26] for tumor and matched normal sample, separately. The detailed format instruction for each required input file is provided in the project website.

We used CONSERTING [27] with default setting to make somatic CNV calls from a dataset of whole-genome sequencing of breast cancer specimens procured in the Roswell Park Cancer Institute (RPCI) (Wei et al., in preparation), and cnvCurator to review the calling results. All study participants provided consent for using their data and specimen for research purposes and the study was approved by Institutional Review Boards at RPCI. The cnvCurator outputs of a CNV call supported by multiple evidences, a missed CNV call, a spurious CNV call, and a CNV call with spurious breakpoint are shown as below. A mini BAM file containing all these exemplar regions is available in the cnvCurator package as demo.

### An example of CNV call supported by multiple evidences

**Fig. 1:**
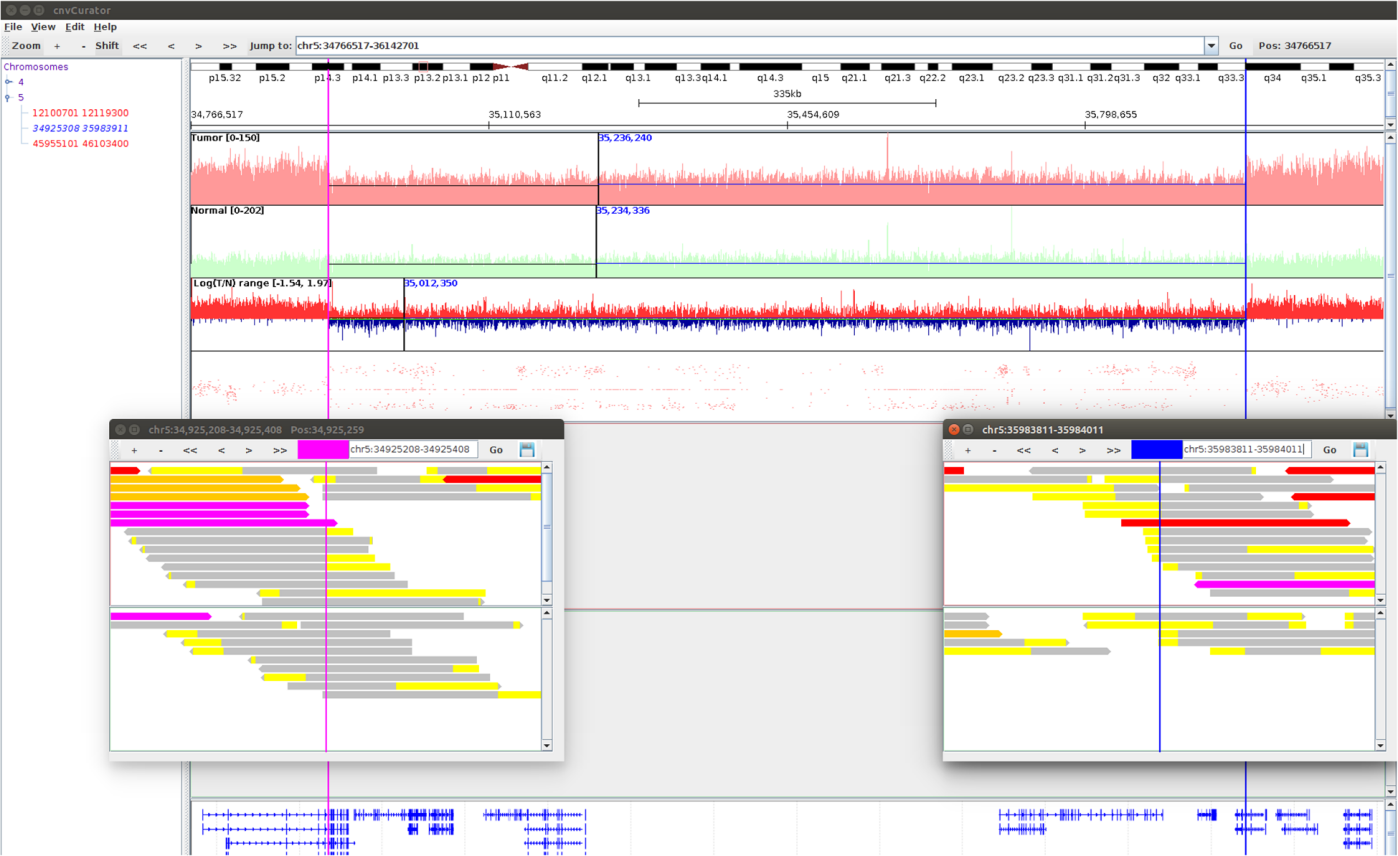
An example of CNV call supported by multiple evidences. The interface of cnvCurator shows segment index on the left and various data tracks on the right. From top to bottom, the tracks include tumor read depth (red line), normal read depth (green line), logR ratio of tumor to normal (red for positive and blue for negative values, respectively), BAF (red dot), read alignment for tumor (in red box), read alignment for normal (in green box), and transcript annotation (blue). The shown region in the main window is base pairs 34766517–36142701 on chromosome 5. The two vertical shading lines (purple on the left, and blue on the right) in the main window delimit the boundary of the called CNV segment (Chr5: 34925308-35983911), with the breakpoint alignments displayed in the two separate pop-up windows. Within each pop-up window, the vertical black line delimitates the exact position of the breakpoint, and the red box and the green box display the tumor bam and the norm bam, separately. For the read displaying, split reads are in yellow–gray with the soft-clipped part in yellow and the matching portion in gray, and the others are discordant read pairs (purple - mate unmapped; red – mate in different chromosome; blue – mate in different strand; orange – mate in discordant distance)

As shown in Fig. 1, this segment of copy number loss is supported by three lines of evidences: 1) decreasing read depth in tumor over normal, as shown in the tracks of read depth and the track of logR ratio of tumor to normal; 2) the corresponding change of B allele frequency (BAF) pattern as shown in the BAF track; 3) the breakpoints are in the exact position separating the soft-clipped part from the matched part of multiple split reads in the tumor bam, but not in the normal bam.

### An example of missed CNV call

**Fig. 2:**
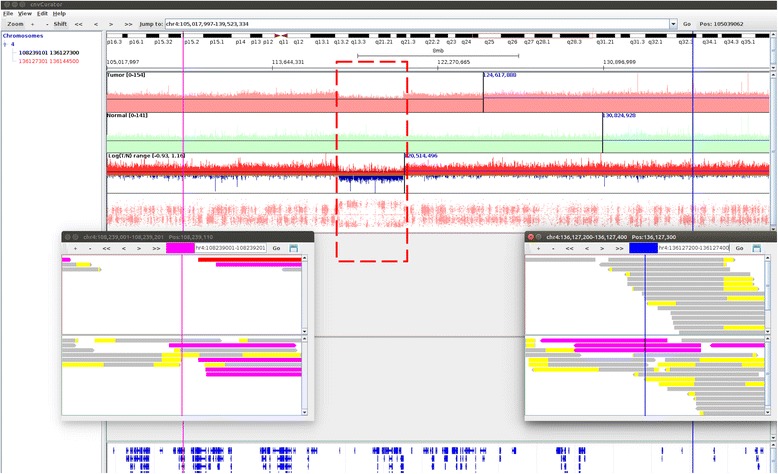
An example of missed CNV call. The shown region in the main window is base pairs 105017997-139523334 on chromosome 4. The two vertical lines (purple on the left, and blue on the right) in the main window delimit the boundary of a segment of copy number neutral (Chr4: 108239101-136127300) called by CONSERTING, with the breakpoint alignments displayed in the two separate pop-up windows. Upon manual review we found this segment contain a sub-segment of copy number loss (marked in rectangle with dashed blue line), as supported by three lines of evidences: 1) decreasing read depth in tumor over normal, as shown in the tracks of read depth and the track of logR ratio of tumor to normal; 2) the corresponding change of BAF pattern as shown in the BAF track; 3) the breakpoints of this sub-segment are in the exact position separating the soft-clipped part from the matched part of multiple split reads in the tumor bam, but not in the normal bam (shown in Fig. 3)

**Fig. 3:**
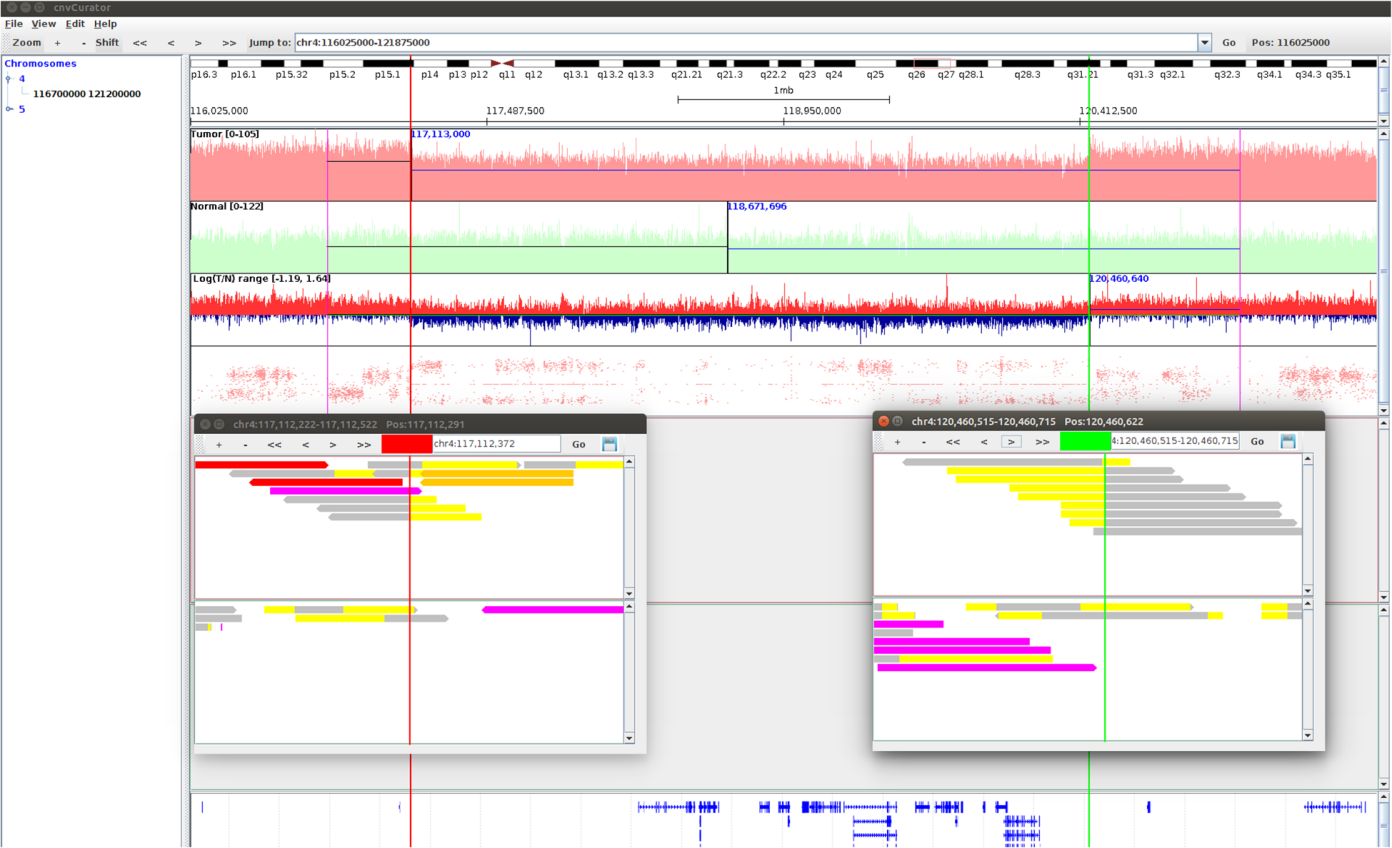
The breakpoint alignment of the missed CNV call. The shown region in the main window is base pairs 116025000-121875000 on chromosome 4. The two vertical lines (red on the left, and green on the right) in the main window delimit the boundary of the segment of copy number loss (Chr4: 117112372-120460622) missed by CONSERTING as described in Fig. 2, with the breakpoint alignments displayed in the two separate pop-up windows

The segment shown in Fig. 2 was called as copy number neutral by CONSERTING. However, upon manual review we found this segment contain a sub-segment of copy number loss, as supported by three lines of evidences: 1) decreasing read depth in tumor over normal, as shown in the tracks of read depth and the track of logR ratio of tumor to normal; 2) the corresponding change of BAF pattern as shown in the BAF track; 3) as shown in Fig. 3, the breakpoints of this sub-segment are in the exact position separating the soft-clipped part from the matched part of multiple split reads in the tumor bam, but not in the normal bam.

### An example of spurious CNV call

**Fig. 4:**
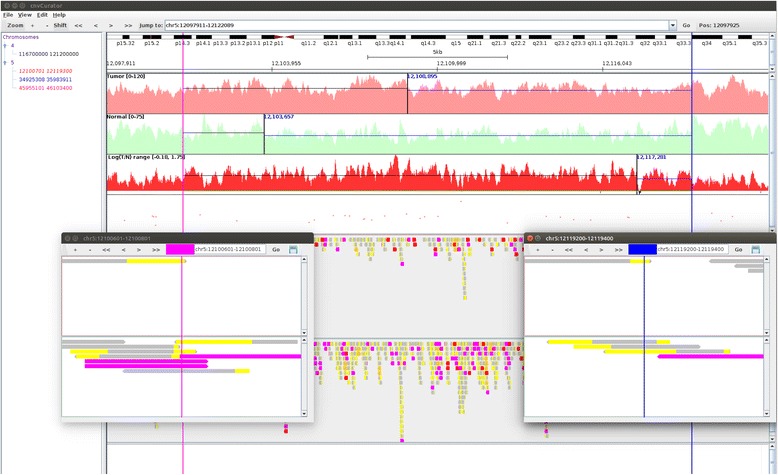
An example of spurious CNV call. The shown region in the main window is base pairs 12097911–12122089 on chromosome 5. The two vertical lines (yellow on the left, green on the right) in the main window delimit the boundary of the spurious CNV call (Chr5: 12100701-12119300), with the breakpoint alignments displayed in the two separate pop-up windows

As shown in Fig. 4, this CNV call is spurious as there is a lack of supporting evidences: 1) there is no clear change of read depth in tumor over normal, as shown in the tracks of read depth and the track of logR ratio of tumor to normal; 2) there is no clear change of BAF pattern as shown in the BAF track; 3) the breakpoint positions are not supported by either split reads or discordant read pairs.

### An example of spurious breakpoint and alternative one

**Fig. 5:**
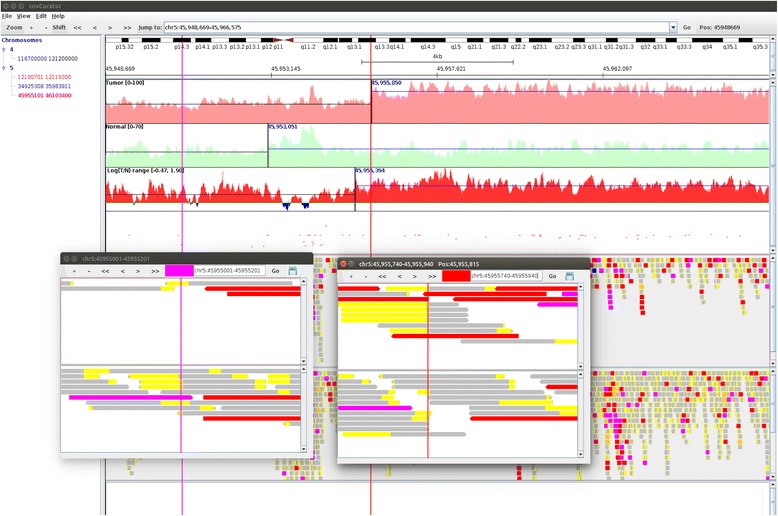
An example of spurious breakpoint and alternative one. The shown region in the main window is base pairs 45948669-45966575 on chromosome 5. The vertical left line (45955101, in purple) in the main window delimits the spurious breakpoint call (Chr5: 45955101), and the vertical right line (45955840, in red) is the alternative breakpoint identified through manual review. The read alignments for the spurious breakpoint in the original call and the alternative breakpoint identified through manual review are displayed in the two separate pop-up windows

As shown in Fig. 5, compared with the spurious breakpoint, the alternative breakpoint is supported by two lines of evidences: 1) the alternative breakpoint is more adjacent to the transition point of read depth changes in tumor over normal, as shown in the tracks of read depth and the track of logR ratio of tumor to normal; 2) the alternative breakpoint is in the exact position separating the soft-clipped part from the matched part of multiple split reads in the tumor bam, and is surrounded by multiple discordant read pairs.

## Discussion

There are several notable differences between cnvCurator and existing genomics viewers, which are summarized as follows: First, as segmentation is a central part of somatic CNV calling methods, the index and display of CNV calling results in cnvCurator is segment based; Second, as boundary prediction is critical for calling CNV (especially focal CNV), cnvCurator provides automatic pop-up windows to examine the detailed breakpoint information; Third, as manual review is often required to curate the somatic variant calling from tumor genome sequencing data, we have implemented several editing functions in cnvCurator to assist in the process of CNV curations. Once the potential segmentation errors are spotted during the manual review, they can be modified through segment splitting/merging functions.

## Conclusions

Due to the special characteristics of tumor samples and the extraordinary complexity of tumor genomes, accurate detection of somatic CNVs is still a great challenge for the community. Here we describe a user-friendly visualization and editing tool specifically designed to facilitate manual review and curation of CNV segments generated by a chosen somatic CNV caller. The viewer provides important and practical utilities to identify problematic CNV segments through manual review, and refine the segmentation results so that user can generate curated results for further analysis.

## Availability and requirements

**Project name:** cnvCurator

**Project home page:**http://www.acsu.buffalo.edu/~lm69/cnvCurator

**Operating system(s):** Windows, Unix-like (Linux, Mac OSX)

**Programming language:** Java

**Other requirements:** Java (TM) 6.0 or higher

**License:** GNU LGPL

**Any restrictions to use by non-academics:** None
